# An optimized graph-based structure for single-cell RNA-seq cell-type classification based on non-linear dimension reduction

**DOI:** 10.1186/s12864-023-09344-y

**Published:** 2023-05-02

**Authors:** Saeedeh Akbari Rokn Abadi, Seyed Pouria Laghaee, Somayyeh Koohi

**Affiliations:** Department of Computer Engineering, Sharif University of Technology, No 717, Tehran, Iran

**Keywords:** scRNA-seq, Clustering, Graph attention autoencoder, Non-linear dimension reduction

## Abstract

**Background:**

It is now possible to analyze cellular heterogeneity at the single-cell level thanks to the rapid developments in single-cell sequencing technologies. The clustering of cells is a fundamental and common step in heterogeneity analysis. Even so, accurate cell clustering remains a challenge due to the high levels of noise, the high dimensions, and the high sparsity of data.

**Results:**

Here, we present SCEA, a clustering approach for scRNA-seq data. Using two consecutive units, an encoder based on MLP and a graph attention auto-encoder, to obtain cell embedding and gene embedding, SCEA can simultaneously achieve cell low-dimensional representation and clustering performing various examinations to obtain the optimal value for each parameter, the presented result is in its most optimal form. To evaluate the performance of SCEA, we performed it on several real scRNA-seq datasets for clustering and visualization analysis.

**Conclusions:**

The experimental results show that SCEA generally outperforms several popular single-cell analysis methods. As a result of using all available datasets, SCEA, in average, improves clustering accuracy by 4.4% in ARI Parameters over the well-known method scGAC. Also, the accuracy improvement of 11.65% is achieved by SCEA, compared to the Seurat model.

## Background

Cells can be differentiated based on the expression of their genes. In contrast to bulk RNA sequencing, which measures gene expression across a large number of samples [1], single-cell RNA sequencing measures gene expression at the cellular level. Single-cell RNA sequencing (scRNA-seq) technology obtains transcriptomics information of cells individually, and it allows the detection of cell types and subtypes at the cellular level [2]. Already, single-cell RNA-sequencing methods have revealed new biology in terms of the composition of tissues, the dynamics of transcription, and the regulatory relationships between genes [3].

The rapid development of single-cell sequencing technologies makes it possible to analyze cellular heterogeneity at the single-cell level [4]. Recently single-cell RNA sequencing has made progress, but still, some challenges remain. For example, weak RNA absorption and the low number of reads in cells are challenges associated with single-cell RNA sequencing protocols and this is reflected as technical zero in the data. Specifically, technical zero normally occurs because of low messenger RNA levels in individual cells, weak absorption, and random expression rates. Consequently, single-cell RNA sequencing data becomes sparse and has high dropout rates. On the other hand, as another challenge, the complex and undetermined distribution of the single-cell RNA sequencing data affects its analysis. Furthermore, the large dimensions of the primary data require efficient methods for dimension reduction. In order to overcome these challenges, in recent years, several methods have been proposed for analyzing single-cell RNA sequencing data taking advantages of deep learning approaches [5–7]. Most of these scRNA-seq pipelines consist of three stages: 1) imputation of dropout events, 2) adoption of dimension reduction methods to identify lower-dimensional representations that explain the maximum variance, 3) Clustering of various cells with similar expressions [8].

Addressing aforementioned goals, Seurat [9], as a popular tools used by biologists, adopts weighted nearest neighbor clustering method, which is used for integrated analysis of multiple data types in a cell. In addition to clustering, Seurat can also be used to infer and analyze single-cell data. On the other hand, many tools have been developed based on deep neural networks for imputation and data reduction. DESC [7] is an Autoencoder-based model that clusters cells and visualizes the results of clustering and gene expression. In DESC, a deep neural network iteratively optimizes a clustering objective function from scRNA-seq data to low-dimensional feature space, and then, it moves each cell to its cluster centroid. The next module is SC3 [10] which presents an interactive clustering tool for scRNA-seq data that is an easy-to-use R package with a graphical interface. The main innovation of SC3 is the demonstration that accurate and robust results can be obtained by combining several well­established techniques using a consensus clustering approach. Through the use of a consensus approach, they are able to achieve high accuracy and robustness. As another analyzing method, ScGNN [6] uses Graph Neural Networks (GNN) [11] for embedding, as well as for cells’ imputation and clustering. Since there is no effective method for graphs denoising, this method does not produce satisfactory results. Similarly, scGAC [5] utilizes a graph neural network, while it adopts a graph denoising approach named Network enhancement (NE) [12]. Specifically, scGAC reduces dimensions in two steps, first Principal Component Analysis (PCA) and then, the graph attention networks are applied. In this manner, it improved the accuracy of single-cell clustering.

Based on the pros and cons of analyzing models presented so far, exploring new methods to improve and increase the accuracy of clustering has been considered in this field of research recently. It is worth noting that although the scGAC model has improved clustering accuracy due to the use of graph attention architecture, adjustment of its various parameters, including the number of head attentions, has not been well investigated. On the other hand, while PCA, as a linear dimension reduction method, is based on the simple assumptions for data analysis, its adoption by scGAC may not be suitable considering the biological data with uncertain distribution [13]. In this regard, a proper non-linear dimension reduction method for single-cell RNA sequencing data should be considered. So, in this work, we propose a new method for clustering single cells RNA sequencing data, named SCEA, which uses two independent units for dimension reduction, as well as a self-optimizing clustering method for cell annotations. For this purpose, a multi-layer perceptron (MLP) based encoder is applied, followed by a GAT [14]. Using eight realistic scRNA-seq datasets as benchmarks, we compare our method with alternative methods in terms of clustering accuracy. Based on the comparative simulation results, we demonstrate that taking advantages of two effective units for dimension reduction, SCEA improves clustering accuracy compared to the baselines. Additionally, SCEA can also be optimized by the Tensor Processing Unit (TPU) architecture, and so, achieves a significant reduction in execution time. It should be noted, our study's primary focus is on dimension reduction, aiming to improve the clustering performance by reducing the data dimensions effectively. For clustering analysis, we utilized the widely used k-means algorithm, which is available in commonly used software packages, making it more accessible and easier to apply in real-world scenarios. Therefore, our main contribution is in the dimension reduction process, which has shown promising results.

## Method and materials

### Method

**Fig. 1:**
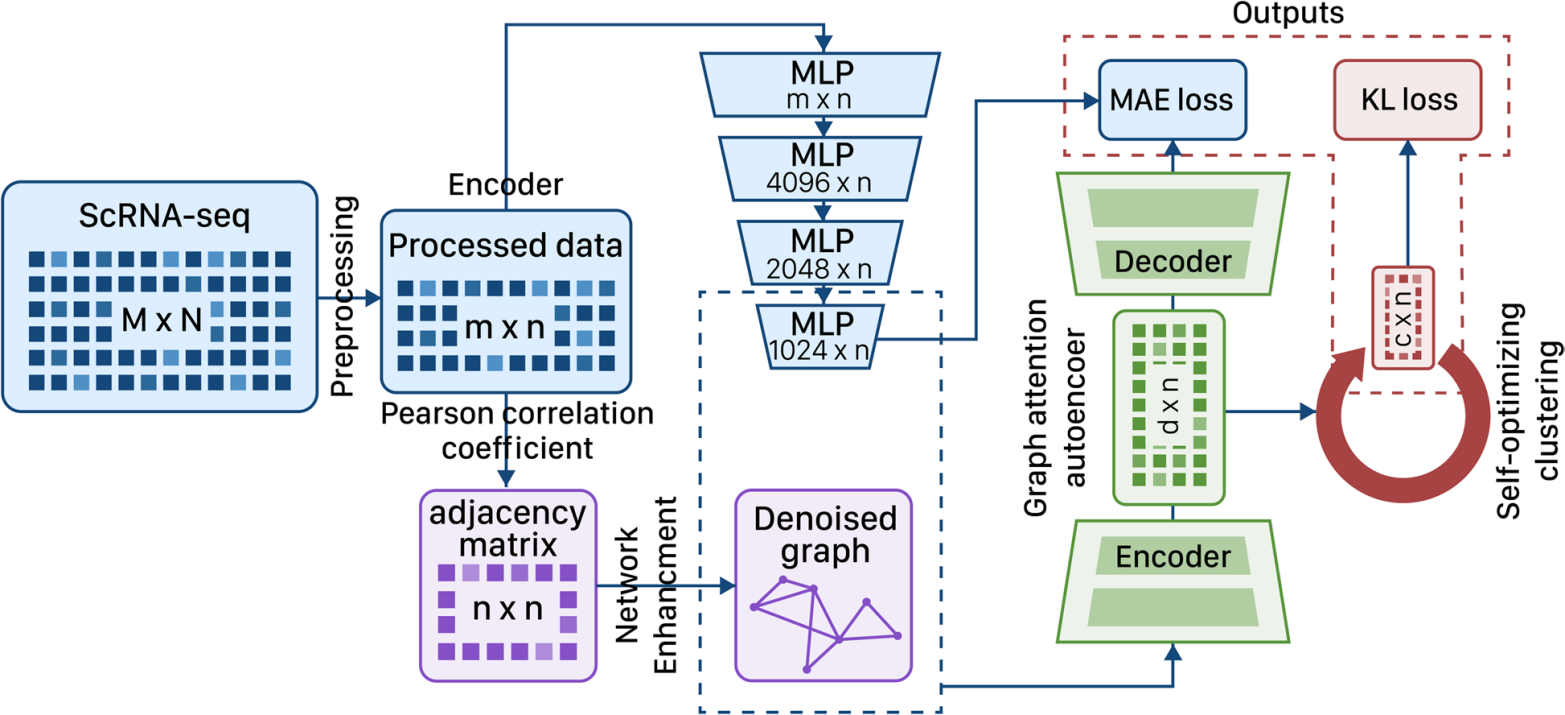
SCEA Workflow, The model consists of a basic MLP neural network with multiple layers and a graph attention neural network used for final dimensionality reduction. The reduced dimensionality of the graph will be used for clustering with the KMeans algorithm. KL loss represents the Kullback Leibler divergence and MAE is the Mean Absolute Error

In this research area, an accurate clustering method should be able to extract important information, such as boundaries and different characteristics among cells, to produce a valid result. In this regard, the SCEA method advances the implementation in four steps, as follows. SECA introduces: a) input data preprocessing, b) graph construction and denoising, c) dimension reduction, d) data clustering by K-means [15]. As depicted in Fig. 1, we first construct a graph using Pearson's correlation coefficient method [16]. At the next step, for graph pruning, we use the Network Enhancement (NE) [12] method which uses a doubly stochastic matrix to find the noisy edge. It is important to emphasize that a square matrix is categorized as doubly stochastic only if all its matrix entries are non-negative, and the sum of the elements in each row and column is equal to one. Out of all the non-negative matrices, stochastic and doubly stochastic matrices hold multiple remarkable properties. To achieve the dimension reduction of data, deep neural networks are used in two steps; as the first step, we use an encoder based on MLP architecture, and then, a graph attention autoencoder [14] uses a cell graph to reduce the dimension of the encoder's output. The graph attention autoencoder, taking advantages of the denoised cell graph containing information on cells connectivity, can extract the connections and bounds between cells, and so, improves the clustering output. As follows, various steps of the proposed method are explained in more details.

#### Step 1: Input data preprocessing

In the proposed method, data preprocessing begins with a raw-count matrix of gene expressions, and so, the SECA tries to filter out the data with poor quality. For this purpose, SECA measures the quality of cells and genes based on their gene expression levels. Once preprocessing is performed, each gene must be expressed in three or further cells. For each cell, SECA determines the expression levels of the appropriate number of genes, as well as the amount of Unique Molecular Identifier)UMI(. In this manner, it can eliminate those with extremely high or extremely low expression levels (based on the first and the third quartiles). For Example, we excluded genes that were expressed in very few cells (e.g., with a zero expression value in most cells) as they may be due to the technical noise or poor sample quality, rather than biological signal. Similarly, we removed genes that had too much expression in most cells, such as housekeeping genes that are constitutively expressed across all cell types and do not contribute to the variance of the data. We determined such genes as having expression values above 75th percentile of the dataset.

#### Step 2: Graph construction and denoising

Once preprocessing is performed, an auxiliary cell graph is constructed to facilitate the information sharing between cells. To construct an initial similarity matrix, the Pearson correlation coefficient [16] between cells is calculated. Then, for graph denoising and achieving more reliable clustering output, the SECA uses NE which takes the adjacency matrix as its input. NE does not alter the eigenvectors while mapping eigenvalues through a non-linear function, and finally, increases the eigengap of the matrix [12]. The diffusion process in NE generates a network consisting of nodes with strong similarity interconnected by edges with large weights, while the nodes with weak similarity are interconnected by edges with small weights. Finally, the Network Enhancement method results an adjacency matrix, while the number of cells connected to each cell is selected based on its k nearest neighbor values. It should be noted, the k-number specifies the number of nearest neighbors that are considered in the cluster formation in the k-nearest neighbors (KNN) algorithm. At the beginning, the algorithm randomly assigns some samples with clusters, which serves as a basis for identifying the rest of the clusters. Each step then updates the center of the clusters, based on the proximity of samples to each other.

#### Step 3: Dimension reduction using neural networks

As the first step of data dimension reduction, SECA uses an MLP-based Encoder. Reducing the dimensions of the data means to condense the data with a large number of features into smaller dimensions. Dimensionality reduction techniques allow us to reduce the complexity and size of datasets, while preserving the variance and discriminative features of the samples. Generally, the single-cell data contains a large number of features (i.e. genes). Encoding, as a nonlinear dimension reduction technique, can reduce data dimensions while preserving its information content. For this purpose, the MPL extracts informational features of the raw data in a low-dimensional space to feed the graph attention autoencoder. The proposed encoder has three layers to reduce data dimension to 4096, 2048, and finally, 1024. Additionally, tanh, as a non-linear activation function [17], is utilized in all layers; so the negative inputs are drawn strongly negative, while the zero inputs are kept near zero. Taking advantages of dimension reduction at the first step, the user can decide whether or not to standardize the output of the encoder.

#### Step 4: Graph attention and clustering

GAT [14] are used to obtain topological information between cells in low dimensions. For this purpose, the cell graph and the encoder's output from the previous step are provided to GAT as its inputs. GAT’s architecture consists of two stacked-graph attentional layers for the encoder and a structurally symmetric decoder. To optimize the graph attention autoencoder, the loss value between the input matrix and the reconstructed matrix is calculated by Mean Absolute Error (MAE) [18]. The multi-head attention [19] can Identify similar cells, as well as the differences between clusters, since it can learn a cell's features by aggregating features of its nearby cells. Accordingly, the attention mechanism can improve clustering results. To perform the clustering task, SECA uses a Self-optimizing clustering module that can optimize the clustering center and redistribute the membership to make the clustering output more sensible. Additionally, the loss value in the proposed method is computed as the combination of MAE for the GAT step and Kullback Leibler Divergence (KLD) [20] for the clustering step.

### Evaluation metrics for clustering

To evaluate the clustering outputs of the proposed method, we use two well-known metrics, Adjusted Rand Index (ARI) [21] and Normalized Mutual Information (NMI) [22] by means of the true dataset labels, as reported in the articles. As shown in Eq. 1 for ARI [21], n_ij_ is the total number of cells that are assigned to the i^th^ cluster according to the model prediction, and assigned to the j^th^ cluster according to the true label. a_i_ is the total number of cells that are assigned to the i^th^ cluster based on the prediction and a_j_ is the total number of cells that are assigned to the j^th^ cluster based on the true label, and finally, n is the total number of clusters.

1 $$ARI=\frac{\sum ij\left(\genfrac{}{}{0pt}{}{{nij}}{2}\right)-\frac{\left[\sum {i }\left(\genfrac{}{}{0pt}{}{{ai}}{2}\right) \sum {j }\left(\genfrac{}{}{0pt}{}{{bj}}{2}\right)\right]}{\left(\genfrac{}{}{0pt}{}{{n}}{2}\right)}}{\frac{1}{2}\left[\sum {i }\left(\genfrac{}{}{0pt}{}{{ai}}{2}\right)+ \sum {j }\left(\genfrac{}{}{0pt}{}{{bj}}{2}\right)\right]-\frac{\left[\sum {i }\left(\genfrac{}{}{0pt}{}{{ai}}{2}\right)+ \sum {j }\left(\genfrac{}{}{0pt}{}{{bj}}{2}\right)\right]}{\left(\genfrac{}{}{0pt}{}{{n}}{2}\right)}}$$

We also use the NMI [22] measure, which is formulated as shown in Eq. 2.

2 $${NMI}\left(X.Y\right)=\frac{\text{I}\left({X.Y}\right)}{2(\mathrm{log}K+\mathrm{log}c)}$$

where, X represents the assigned clustering, Y represents the pre-existing labels on the same data, k is the number of clusters, c is the number of pre-existing classes, and finally, *I* (X. Y) calculate the mutual information between X and Y, as formulated in Eq. 3

3 $${I} \left({X}.{Y}\right)={H} \left({X. Y}\right)-( ({H}\left({X|Y}\right)+{H}\left({Y|X}\right))$$

where, H(Z) calculates the marginal information entropy, H(X|Y) represents the conditional entropy, and H (X. Y) calculates the joint entropy.

### Datasets

We evaluate SECA on a diverse set of challenging single-cell datasets (Klein [23] (GSE65525), Zeisel [24] (GSE60361), Romanov [25] (GSE74672), Chung [26] (GSE75688), Pbmc [27] (https://support.10xgenomics.com/single-cell-gene-expression/datasets/2.1.0/pbmc4k), Mouse [28] (https://figshare.com/s/865e694ad06d5857db4b), Biase [29] (GSE57249), Petropoulos [30] (https://www.ebi.ac.uk/arrayexpress/exp), Neurons_5K [31] (https://cf.10xgenomics.com/samples/cell-exp/6.0.0/SC3_v3_NextGem_DI_Neurons_5K_SC3_v3_NextGem_DI_Neurons_5K/SC3_v3_NextGem_DI_Neurons_5K_SC3_v3_NextGem_DI_Neurons_5K_web_summary.html), Mouse Brain [32] (https://www.10xgenomics.com/resources/datasets/mouse-tissue-microarray-in-3x3-layout-with-2-mm-edge-to-edge-spacing-ffpe-2-standard)) that range from humans to mice, as listed in Table 1. Gene’s counts range from 15,344 to 27,420 and cells are from 49 to 5483. We cover data with the different numbers of cells in our dataset package. We have small data like biase which has 49 cells and large data like Pbmc which has 4220 cells. We also used the scanpy [33] tool to extract the expression matrix from the existing feature/barcode matrix for the Neuron data and Mouse brain data.

**Table 1:** Dataset description and details

Dataset	Type	# of cluster	# of cell	# of gene	Seq platform
Klein	Homo sapiens	4	2717	24,021	inDrop
Zeisel	Mus musculus	7	2998	18,869	Illumina HiSeq
Romanov	Mus musculus	7	2863	18,496	Illumina HiSeq
Chung	Homo sapiens	5	515	27,420	Illumina HiSeq
Pbmc	Homo sapiens	8	4220	16,412	10X
Mouse	Mus musculus	16	2044	15,344	Microwell-seq
Biase	Mus musculus	3	49	21,489	Illumina HiSeq
Petropoulos	Homo sapiens	5	1518	21,627	Illumina HiSeq
Neuron	Homo sapiens	11	5483	32,286	cell ranger
Mouse Brain	Mus musculus	5	501	19,465	Space Ranger

## Results

As discussed in the previous section, our clustering architecture includes some metadata and steps, and so, as follows, we investigate their impact on the clustering accuracy. For this purpose, the following assessments are established:

1. The impact of standardization on the SCEA’s performance.
2. The impact of the number of head attentions on the SCEA’s performance.
3. The impact of nonlinear dimension reduction, compared to the linear type (like PCA), on the SCEA’s performance.

To determine the accuracy of clustering, we use two parameters, ARI [21] and NMI [22]. We also examine how the TPU affects run time, as shown in the following section.

### Analyzing the impact of standardization

**Fig. 2:**
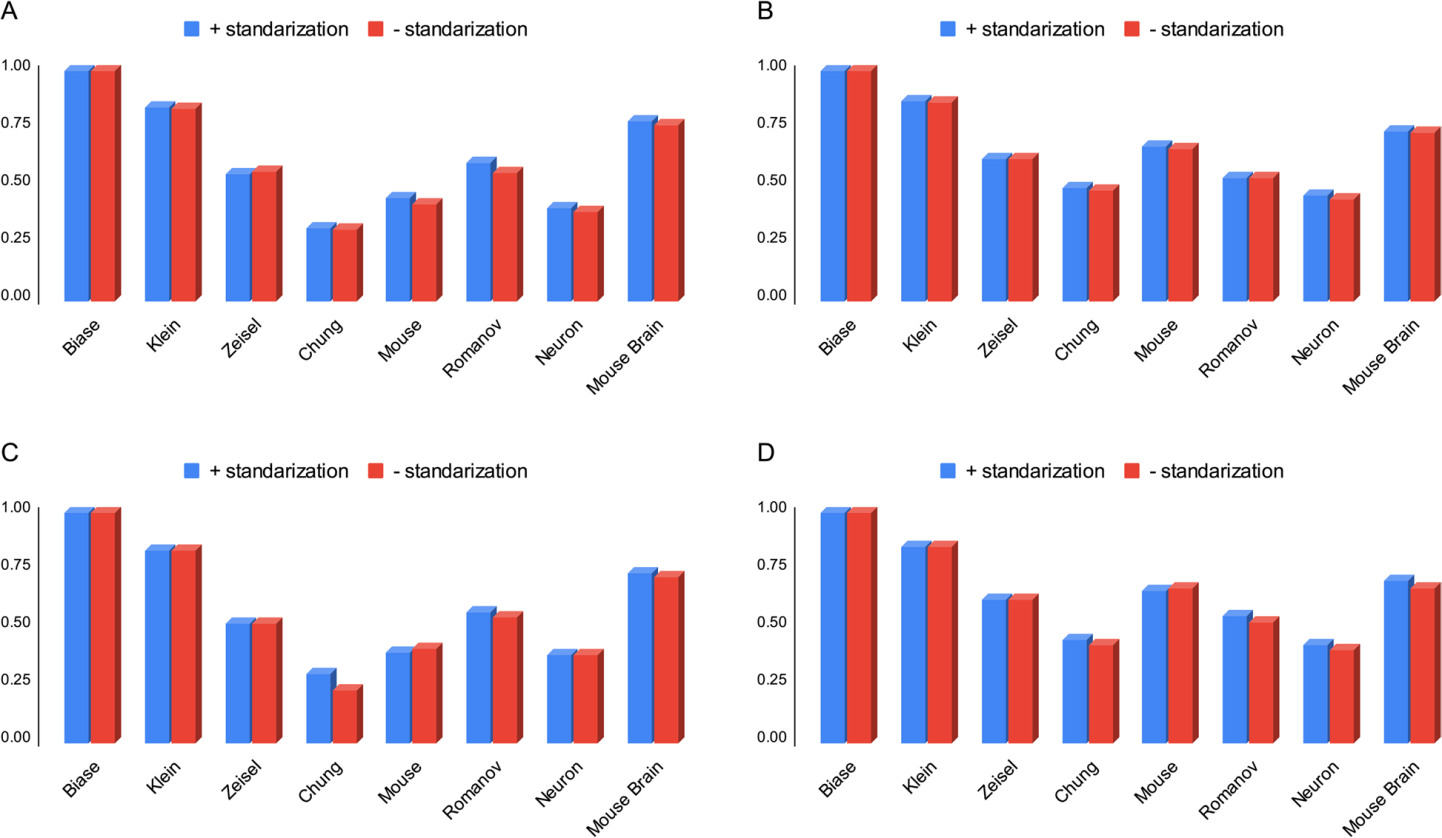
Analysis of the effect of standardization on the clustering accuracy of SCEA and scGAC. **A** investigate the impact on SCEA accuracy using the parameter ARI. **B** investigate the impact on SCEA accuracy using the parameter NMI. **C** investigate the impact using the parameter ARI on scGAC. **D** investigate the impact using the parameter NMI on scGAC

Using standardization to contain data values in the fixed range can improve their applicability in many data analyzing applications. Therefore, in this section, we assess the impact of standardization, as defined in Eq. 4, on the accuracy of cell typing. Figure 2 shows the impact of standardization on the accuracy of cell typing. The plot compares the performance of two different algorithms, SCEA and scGAC, on eight different datasets using standardized and non-standardized data. The results indicate that standardization improves the accuracy of cell typing in seven out of eight datasets, as evidenced by higher values of NMI [22] and ARI [21]. However, it is important to note that this improvement is only slight. Overall, this suggests that standardization can be a useful tool for improving the accuracy of cell typing, but it may not have a significant impact on performance in all cases. It is up to the user to decide whether to use standardization based on the specific requirements of their analysis. Nevertheless, SCEA considers the standardization as an option which can be activated by the user. For brevity, in the following, SCEA with and without the standardization is specified as SCEA_s_ and SCEA_ns_, respectively. It is worth noting that aside from the NMI and ARI reports, we have also disclosed the *p*-value results of our model for the datasets accessible through our project link. All datasets have generated *p*-values below 0.05, thereby establishing the credibility of both modes.

4 $$\mathrm{X}\left(\mathrm{scaled}\right)=\frac{\mathrm{X}-\mathrm{mean}}{sd}$$

### Analyzing the impact of the number of head attentions

While using a multi-head attention mechanism [19] in our architecture, the number of head attentions can affect the SCEA’s performance, as discussed in this section. For this purpose, we examine five different numbers of head attentions: two, four, six, eight, and ten. To achieve a comprehensive statement, five data sets are considered for this analysis: Biase [29], Chung [26], Mouse [28], Petropoulos [30], and Mouse Brain. We would like to clarify the rationale behind our choice of range for the number of headers in our model. Firstly, in similar works [5, 34] that have employed the use of multi-head attention, values of 4 and 8 have commonly been selected. We have also chosen to explore two, four, six, eight, and ten as the number of headers, based on the success of these previous studies. Secondly, it should be noted that increasing the number of headers can result in higher computational overhead without a significant improvement in performance. Therefore, we have limited the range to a maximum of 10, in order to maintain practical usability and avoid unnecessary computational burden.

**Fig. 3:**
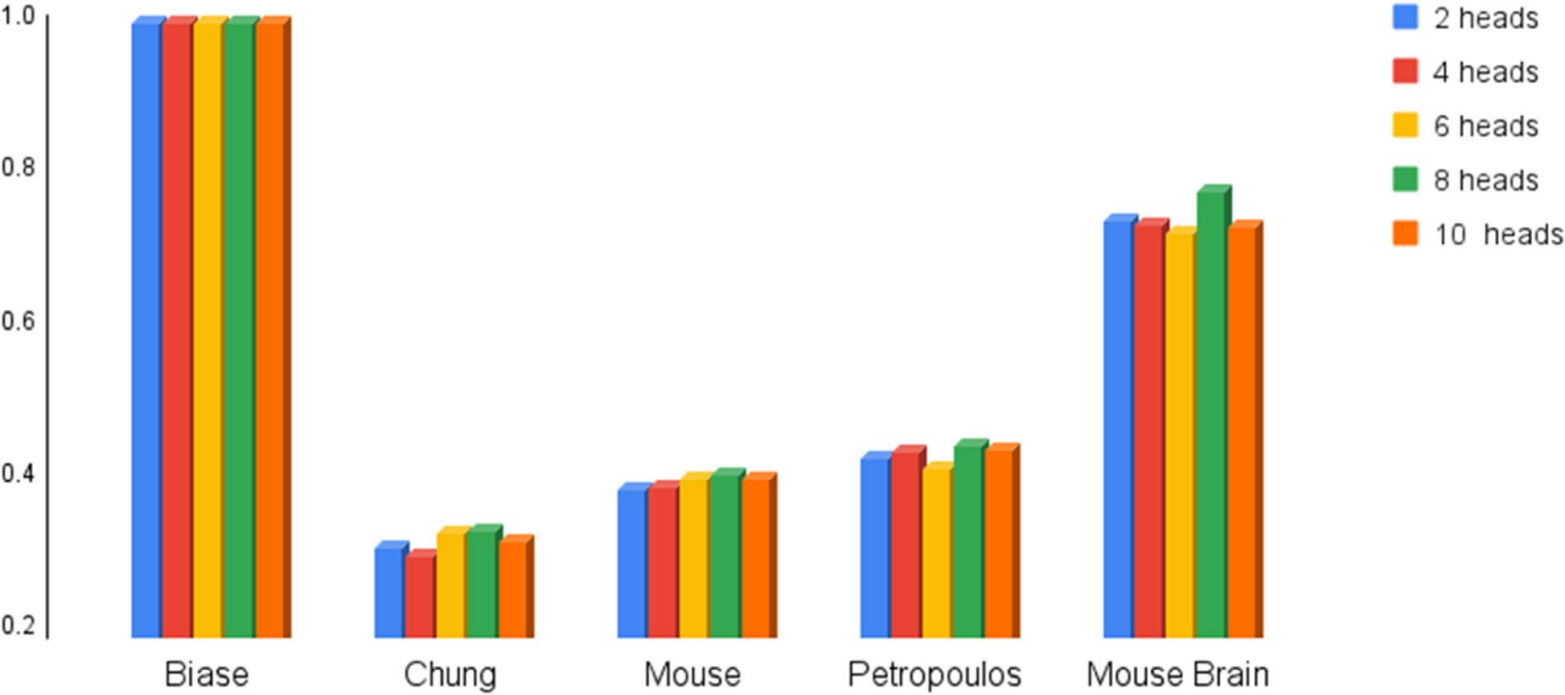
Analysis of ARI value for different numbers of head attention

**Fig. 4:**
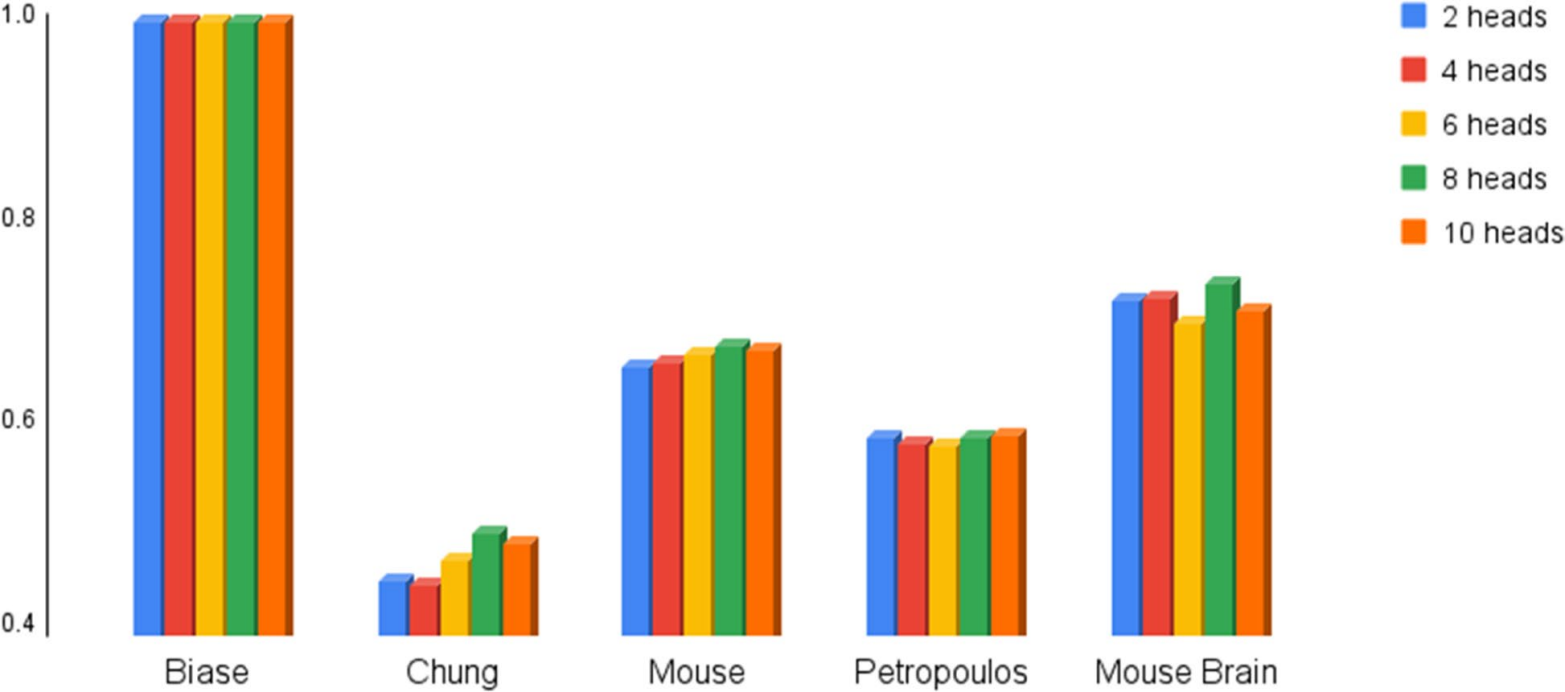
Analysis of NMI value for different numbers of head attention

As shown in Figs. 3 and 4, eight number of head attentions leads to a minor increase in both ARI [21] and NMI [22] in comparison to other cases, while taking advantages of 10 head attentions has no significant impact on the SCEA’s accuracy. On the other hand, since increasing this parameter results in a larger execution time, its minimization should be considered. Finally, it is worth noting that taking advances of TPUs facilitates runtime management, and so, extra runtime resulted from the multi-head attentions can be afforded. It is noteworthy that gene expression data has an unknown distribution, making it challenging to select the optimal number of head attention. To tackle this issue, we conducted numerous tests with different combinations of parameters, and we found that the best value for the number of head attentions is eight. We implemented this parameter on various datasets (from 49 to 5000 cells) and observed that it consistently provided the best results with a small difference in all cases. Therefore, we conclude that the selected parameter (i.e., eight head attentions) is optimal for our problem, considering the designed structure and the number of cells in our investigations. It should be noted that the results of this experiment are also given in Table 2.

**Table 2:** ARI and NMI value for different numbers of head attention

	**2 head**		**4 head**		**6 head**		**8 head**		**10 head**	
**Dataset**	**ARI**	**NMI**	**ARI**	**NMI**	**ARI**	**NMI**	**ARI**	**NMI**	**ARI**	**NMI**
Biase	100	100	100	100	100	100	100	100	100	100
Chung	31.15	44.9	30.3	44.6	33.19	47.04	33.40	49.6	32.22	48.73
Mouse	39.00	65.98	39.31	66.43	40.36	67.31	40.82	68.05	40.16	67.6
Petropoulos	42.99	59.12	43.93	58.39	41.75	58.17	44.50	59.09	44	59.19
Mouse Brain	74.1	72.5	73.6	72.8	72.33	70.33	77.8	74.1	73.33	71.5

### Analyzing the impact of nonlinear dimension reductions

When working with large datasets, dimension reduction is inevitable to facilitate runtime management. Dimension reduction refers to the transformation of high-dimensional data into low-dimensional data to retain some meaningful properties of the original data, ideally close to its intrinsic dimensions [35]. In scGAC, PCA is used as the main linear technique for dimension reduction that maps data to a smaller space in such a way that it maximizes the variance of the data. However, it might be leaving out features that do not explain much of the variance of the dataset but do explain what characterizes one class against another. For PCA to be effective, data elements must be correlated, otherwise, it performs poorly on uncorrelated data [13].

**Fig. 5:**
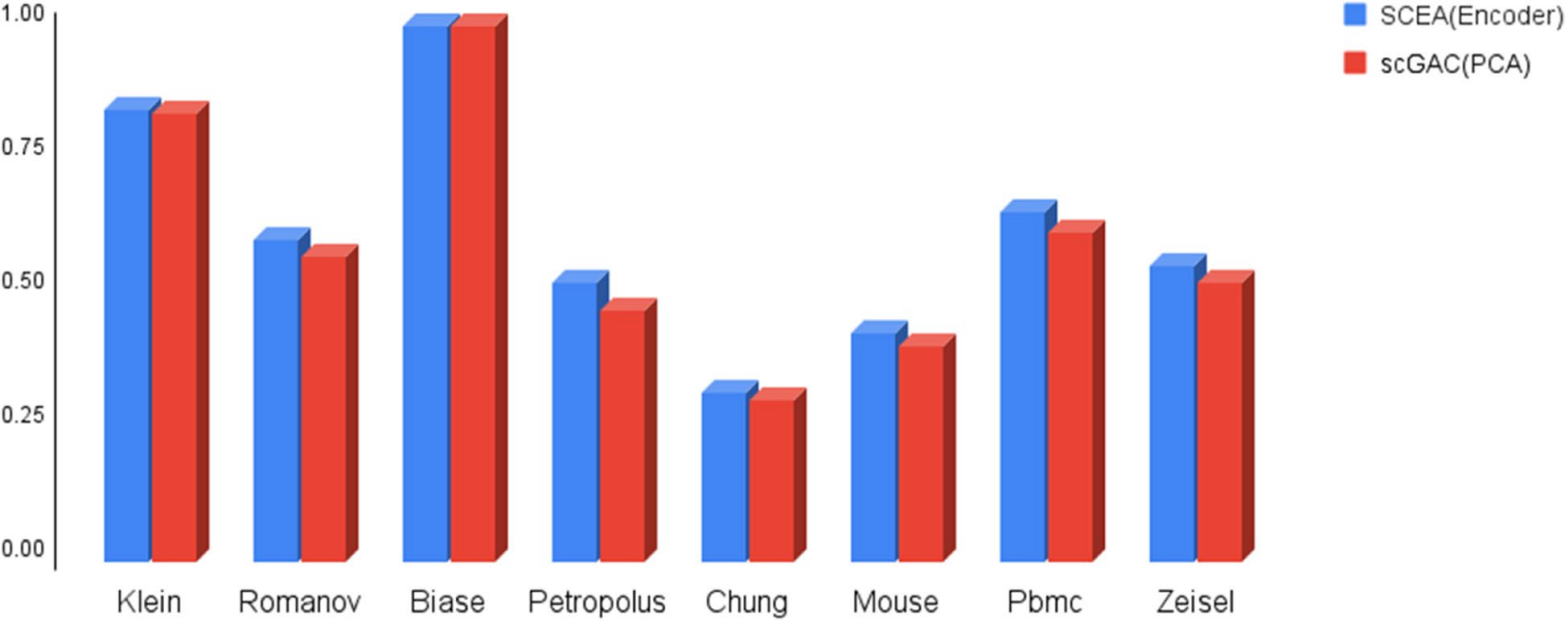
Analyzing the ARI value in order to compare different methods of dimension reduction

**Fig. 6:**
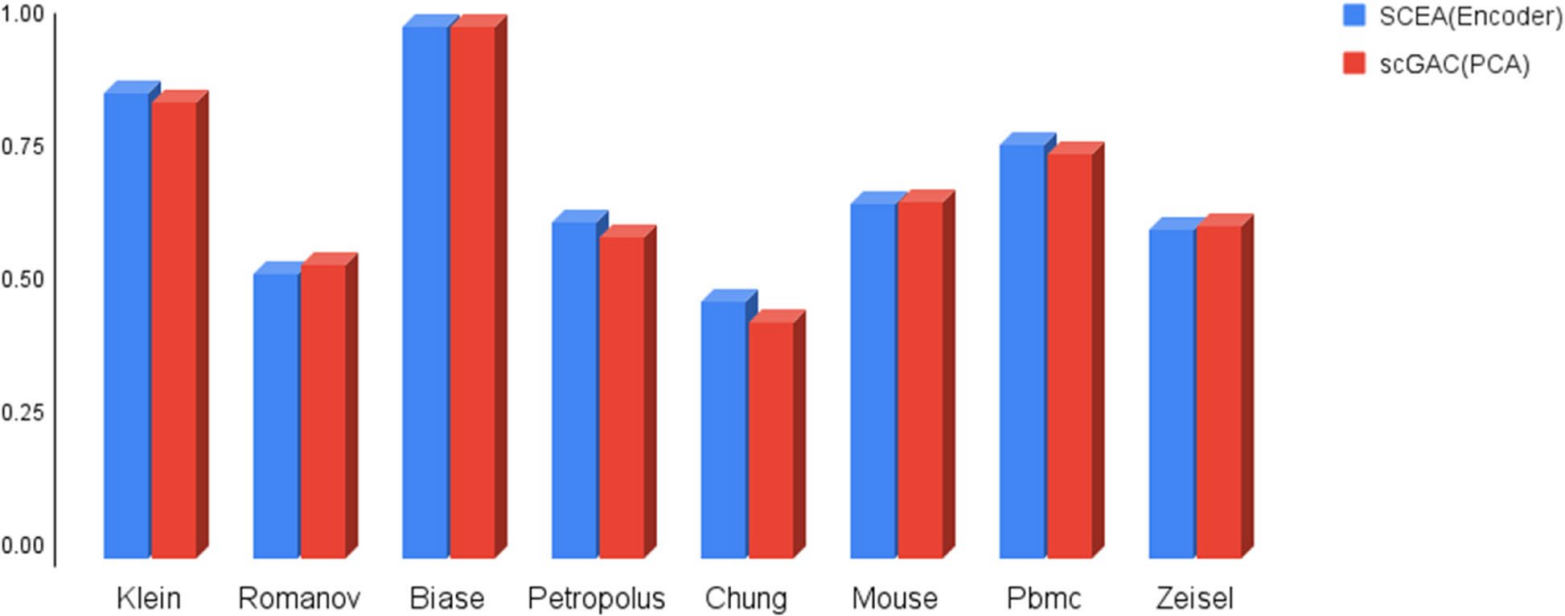
Analyzing the NMI value in order to compare different methods of dimension reduction

Considering that the biological data have an indeterminate and complex distribution, and the relationships among features may not have a linear factorization, it is more appropriate to use nonlinear dimension reduction techniques. In this manner, we proposed an encoder network with layers of MLP and reduced the data dimensions to 1024, as shown in Fig. 1. We also used tanh as a non-linear activation function in each layer [17]. To investigate the capability of the proposed dimension reduction network, we compare it against scGAC [5], which employs a linear method of dimension reduction, PCA. As shown in Figs. 5 and 6, the proposed non-linear dimension reduction network improves the clustering outputs in eight benchmarks.

In the next step, based on the results of the previous sections, we use the most optimal possible conditions for SCEA. In other words, SCEA accuracy refers to the accuracy obtained from the best experience including:

1. To reduce dimensions in the first step, we use an encoder based on Tensorflow.
2. During the dimension reduction process, we also use a Graph attention Autoencoder [14] with a set of eight head attentions.
3. We use the standardization option for SCEA.

**Fig. 7:**
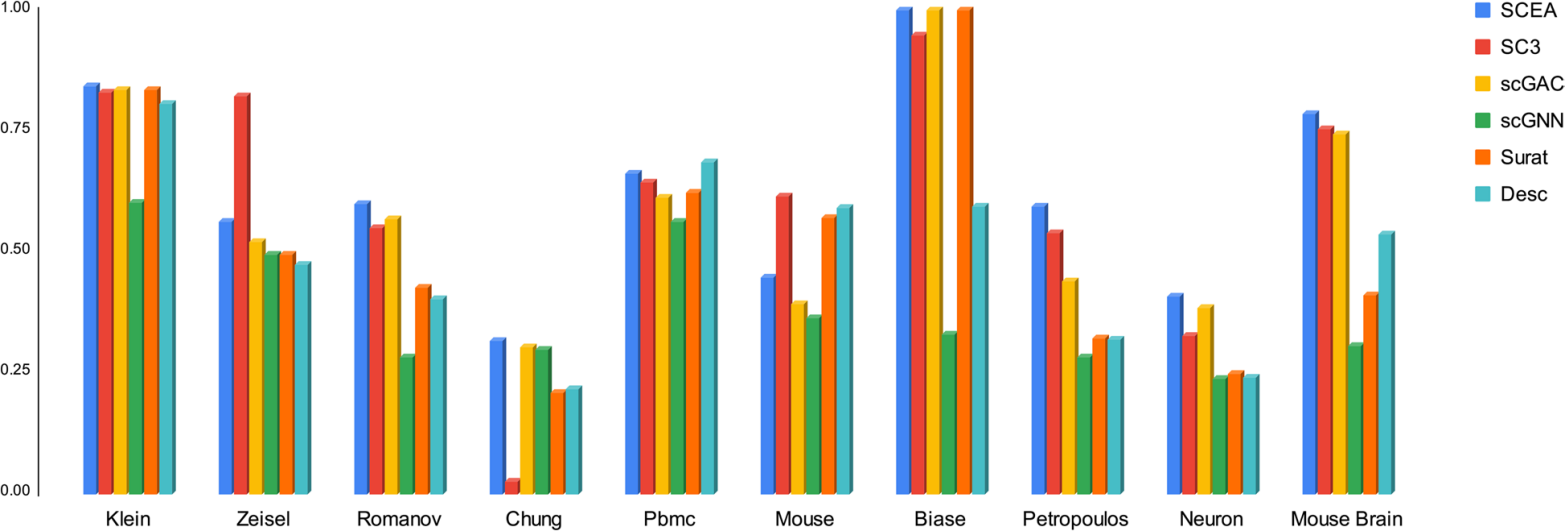
Comparison of Adjusted rand index (ARI) for baselines and SCEA

**Fig. 8:**
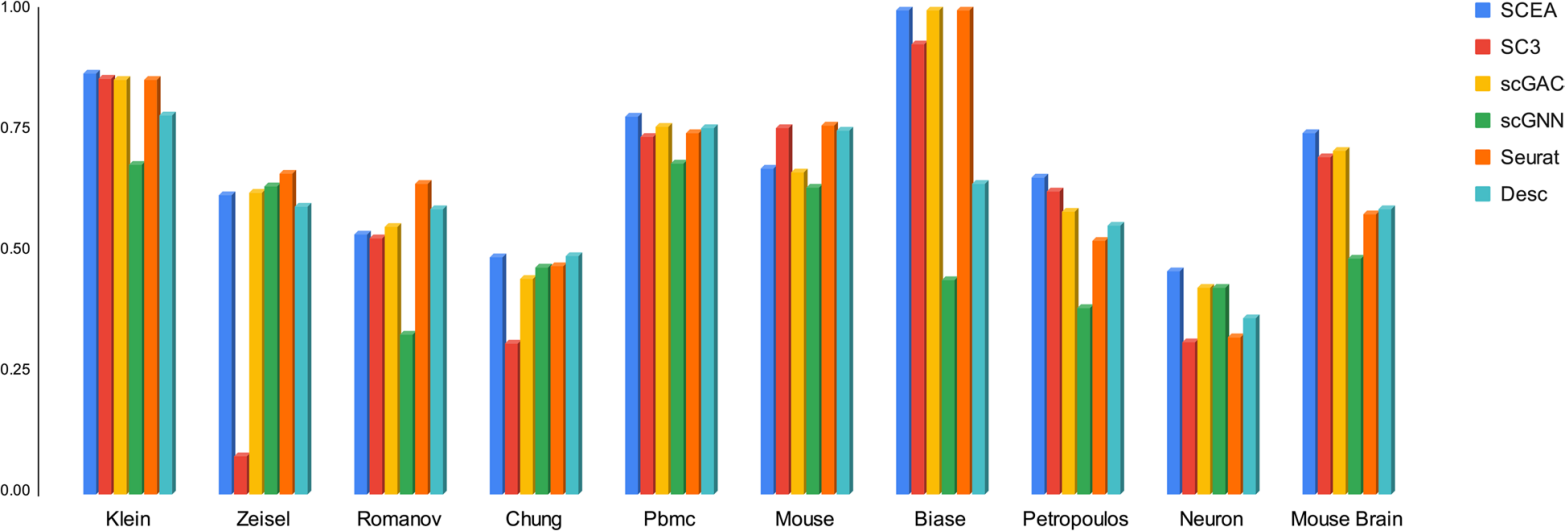
Comparison of Normalized mutual information (NMI) for baselines and SCEA

As reported in Figs. 7 and 8, SCEA achieves the best accuracy, compared to the four alternative models in terms of two parameters, ARI [21] and NMI [22]. Detailed information regarding the accuracy of the models can be found in the Table 3.

**Table 3:** Simulation result for all compared methods

	**SCEA**		**SC3**		**scGAC**		**scGNN**		**Seurat**		**Desc**	
Dataset	**ARI**	**NMI**	**ARI**	**NMI**	**ARI**	**NMI**	**ARI**	**NMI**	**ARI**	**NMI**	**ARI**	**NMI**
Klein	**0.843**	**0.869**	**0.831**	**0.859**	**0.835**	**0.856**	**0.601**	**0.680**	**0.836**	**0.856**	**0.808**	**0.784**
Zeisel	**0.562**	**0.616**	**0.822**	**0.076**	**0.520**	**0.624**	**0.495**	**0.635**	**0.494**	**0.661**	**0.473**	**0.593**
Romanov	**0.600**	**0.535**	**0.551**	**0.530**	**0.569**	**0.553**	**0.283**	**0.330**	**0.426**	**0.641**	**0.403**	**0.589**
Chung	**0.317**	**0.490**	**0.025**	**0.310**	**0.303**	**0.446**	**0.298**	**0.469**	**0.209**	**0.470**	**0.215**	**0.493**
Pbmc	**0.661**	**0.781**	**0.643**	**0.737**	**0.613**	**0.760**	**0.562**	**0.683**	**0.623**	**0.746**	**0.687**	**0.757**
Mouse	**0.446**	**0.672**	**0.616**	**0.757**	**0.393**	**0.664**	**0.362**	**0.635**	**0.571**	**0.762**	**0.593**	**0.752**
Biase	**1.00**	**1.00**	**0.948**	**0.929**	**1.00**	**1.00**	**0.330**	**0.443**	**1.00**	**1.00**	**0.594**	**0.641**
Petropoulos	**0.594**	**0.654**	**0.538**	**0.627**	**0.439**	**0.583**	**0.282**	**0.384**	**0.322**	**0.523**	**0.318**	**0.555**
Neuron	**0.408**	**0.461**	**0.327**	**0.315**	**0.385**	**0.427**	**0.236**	**0.425**	**0.249**	**0.324**	**0.239**	**0.364**
Mouse Brain	**0.786**	**0.746**	**0.754**	**0.696**	**0.744**	**0.708**	**0.306**	**0.486**	**0.410**	**0.579**	**0.535**	**0.588**

### Analyzing the impact of TPU on runtime

TPUs are Google's custom-developed Application-Specific Integrated Circuits (ASICs) used for accelerating machine learning workloads. Researchers, developers, and businesses can leverage TensorFlow computing clusters that use Cloud TPU for maximum performance and flexibility. It should be clarified that TPU is not included in our algorithm. Instead, since a portion of our model was constructed using the TensorFlow package, we explored the potential of utilizing TPU to enhance the computational speed. Typically, when dealing with extensive matrix operations, executing the code on a TPU can greatly enhance the speed of computation compared to a CPU. However, it should be noted that not all types of code can be optimally accelerated through TPUs. In instances, where the code has minimal computational intensity or contains numerous branches or conditional statements, the TPU may not offer notable gains in terms of computational speed compared to a CPU or GPU. Furthermore, if the code necessitates significant memory bandwidth, TPU may not be the optimal choice, owing to their focus on computation as opposed to memory access. Based on our code's description, it exhibits exceptional performance when executed on TPU, highlighting its developmental benefits.

**Fig. 9:**
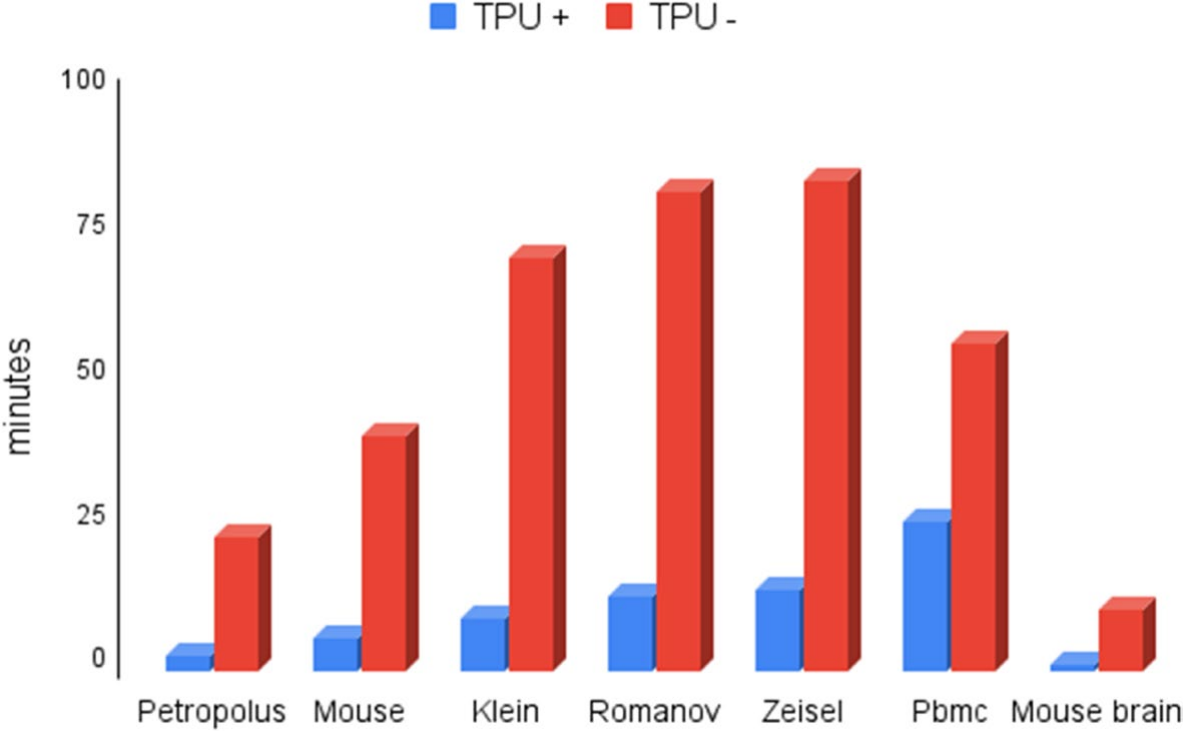
Comparison of execution times to find the effect of TPU on 7 datasets

At the first step of dimension reduction, as shown in Fig. 1, we propose utilization of an encoder based on Tensorflow. Figure 9 illustrates various experiments to investigate whether TPU can improve runtime of different applications. We have used the TPU version3 available in colab [36] with the configuration of 36 RAM, and 10 GB cache.

As shown in Fig. 9, runtime of pre-training process increases exponentially in the non-TPU execution mode. However, this figure shows a significant runtime improvement for TPU-base implementation over the non-TPU one. It is worth noting that we examines the scGAC model in a similar manner, and concluded that taking advantage of TPU cannot improve its runtime significantly. Table 4 presents the results of all tests.

**Table 4:** Duration of each experiment in two modes of using and not using the TPU (based on minutes)

Dataset	+ TPU	-TPU
Petropolus	2.78	23.18
Mouse	5.98	40.93
Klein	9.35	71.52
Romanov	12.95	82.72
Zeisel	14.13	84.89
Pbmc	26.08	56.58
Biase	0.36	0.55
Mouse_brain	1.26	10.91
Chung	0.8	3.35

**Fig. 10:**
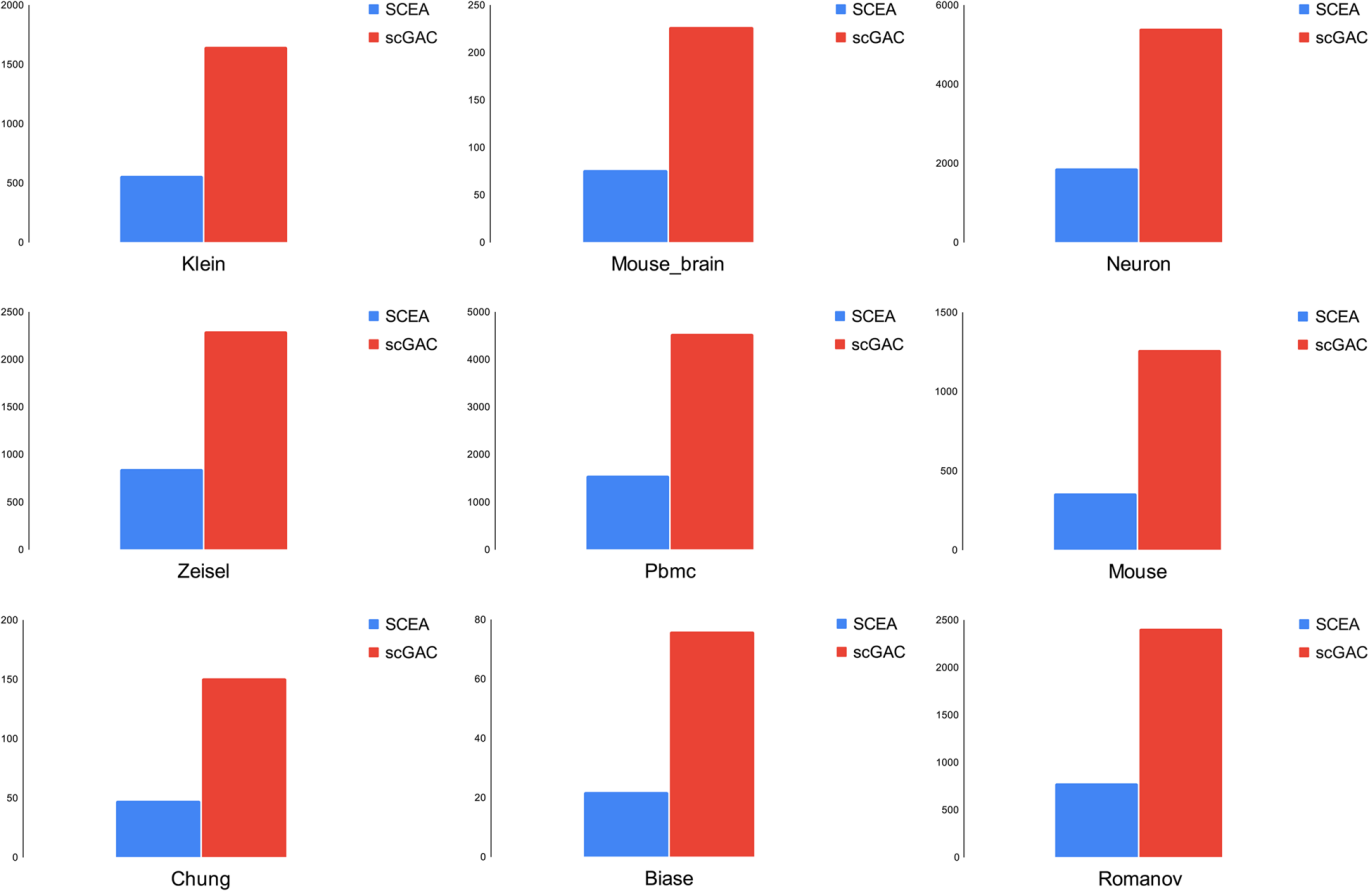
comparison of execution time of SCEA with scGAC on 9 Datasets (klein, Mouse brain, Neuron, Zeisel, Pbmc, Mouse, Chung, Biase, Romanov). The y-axis line is based on seconds

To evaluate our proposed model's efficiency, we compared it to the scGAC model across all datasets with respect to the execution time. We conducted these comparisons to determine whether our method produced results more quickly than scGAC, which is considered to be one of the standard baseline methods. The comparison process was performed using a range of datasets, of varying sizes and complexities. We recorded the time taken by both models to process these datasets and drew insights from the results. The findings are visually represented in Fig. 10, which provides a graphical comparison of our proposed method's execution time against scGAC.

When examining the results, it was observed that our approach was indeed faster than scGAC. Our method was able to generate results in all datasets more quickly, thus providing a more efficient method for processing large datasets. Additionally, we observed that our proposed model was able to complete the processing task while using significantly fewer computational resources, making it a more cost-effective option for processing large-scale datasets.

## Discussion and conclusion

To identify cell identities and functions using scRNA-seq data, it is necessary to cluster different cells according to their gene expression. In this study, using the scGAC [5] tool, we have developed a method called SCEA that gives us the best accuracy for clustering among famous and reliable models. The complex and irregular distribution of single-cell RNA-seq data is one of the main challenges for cells clustering. In scGAC [5], although adoption of PCA based on the simple assumptions reduces hardware costs and execution times, does not perform well for dimension reduction of single-cell data. Therefore, to address this drawback, we propose adoption of an encoder neural network, which applies a non-linear reduction of dimensionality. In addition, we realized that increasing the number of head attentions can improve accuracy (up to a certain extent). Moreover, by using TPU, we have shown that the execution time can be limited. Specifically, for approximately 5000 cells, the execution time will be less than 30 min. Our method also includes two modes, considering either standardization or non-standardization of the dimensionally reduced data produced by the encoder. Although either choice is applicable, we suggest using a method with data standardization, since based on our simulation results, it improves the clustering accuracy. Simulating eight realistic scRNA-seq datasets as benchmarks, we show that SCEA can outperform state-of-the-art methods in scRNA-seq clustering.

Future improvements can be made in several directions. Efficient attention-based models, such as transformers instead of GAT [14], which are also something we follow seriously. The second issue is improving noise removal conditions in the cell graphs would be considered to significantly improve the final result. Finally, as we concluded, valid biological concepts discovered so far, such as protein–protein interaction networks, can be integrated into the model to precisely determine the state of communication between cells.

## Data Availability

Source code and data are freely available for download at https://github.com/SAkbari93/SCEA.git, implemented in python, and supported on Linux and MS Windows.

## Abbreviations

scRNA-seq: Single-cell RNA sequencing
GNN: Graph Neural Networks
NE: Network Enhancement
MLP: Multi-Layer Perceptron
TPU: Tensor Processing Unit
PCA: Principal Component Analysis
MAE: Mean Absolute Error
UMI: Unique Molecular Identifier
KLD: Kullback Leibler Divergence
ARI: Adjusted Rand Index
NMI: Normalized Mutual Information
ASIC: Application-Specific Integrated Circuit
